# Developmental Toxicity and Biotransformation of Two Anti-Epileptics in Zebrafish Embryos and Early Larvae

**DOI:** 10.3390/ijms222312696

**Published:** 2021-11-24

**Authors:** Chloé Bars, Jente Hoyberghs, Allan Valenzuela, Laura Buyssens, Miriam Ayuso, Chris Van Ginneken, Alain J. Labro, Kenn Foubert, Steven J. Van Cruchten

**Affiliations:** 1 Comparative Perinatal Development, Department of Veterinary Sciences, Faculty of Pharmaceutical, Biomedical and Veterinary Sciences, University of Antwerp, Universiteitsplein 1, 2610 Wilrijk, Belgium; Chloe.bars@uantwerpen.be (C.B.); Jente.hoyberghs@uantwerpen.be (J.H.); Allan.valenzuela@uantwerpen.be (A.V.); Laura.buyssens@uantwerpen.be (L.B.); Miriam.ayusohernando@uantwerpen.be (M.A.); Chris.vanginneken@uantwerpen.be (C.V.G.); 2 Laboratory of Molecular, Cellular and Network Excitability, Department of Biomedical Sciences, University of Antwerp, Universiteitsplein 1, 2610 Wilrijk, Belgium; Alain.labro@uantwerpen.be; 3 Department of Basic and Applied Medical Sciences, Faculty of Medicine and Health Sciences, Ghent University, Corneel Heymanslaan 10, 9000 Ghent, Belgium; 4 Natural Products and Food Research and Analysis (NatuRA), Department of Pharmaceutical Sciences, University of Antwerp, Universiteitsplein 1, 2610 Wilrijk, Belgium; Kenn.foubert@uantwerpen.be

**Keywords:** developmental toxicology, in vitro, zebrafish, embryo, drug metabolism, bioactivation

## Abstract

The zebrafish (*Danio rerio*) embryo is gaining interest as a bridging tool between in-vitro and in-vivo developmental toxicity studies. However, cytochrome P450 (CYP)-mediated drug metabolism in this model is still under debate. Therefore, we investigated the potential of zebrafish embryos and larvae to bioactivate two known anti-epileptics, carbamazepine (CBZ) and phenytoin (PHE), to carbamazepine-10,11-epoxide (E-CBZ) and 5-(4-hydroxyphenyl)-5-phenylhydantoin (HPPH), respectively. First, zebrafish were exposed to CBZ, PHE, E-CBZ and HPPH from 5¼- to 120-h post fertilization (hpf) and morphologically evaluated. Second, the formations of E-CBZ and HPPH were assessed in culture medium and in whole-embryo extracts at different time points by targeted LC-MS. Finally, E-CBZ and HPPH formation was also assessed in adult zebrafish liver microsomes and compared with those of human, rat, and rabbit. The present study showed teratogenic effects for CBZ and PHE, but not for E-CBZ and HPPH. No HPPH was detected during organogenesis and E-CBZ was only formed at the end of organogenesis. E-CBZ and HPPH formation was also very low-to-negligible in adult zebrafish compared with the mammalian species. As such, other metabolic pathways than those of mammals are involved in the bioactivation of CBZ and PHE, or, these anti-epileptics are teratogens and do not require bioactivation in the zebrafish.

## 1. Introduction

In the last two decades, the field of toxicology has put a strong emphasis on the exploration and development of in-vitro models that accurately predict human toxicity. Developmental toxicity studies are assessing embryofetal death, reduced fetal growth and abnormal development following xenobiotic exposure in laboratory animals [1]. However, several initiatives have been set-up to reduce animal testing and to comply with the 3R’s principle. This has resulted in openings for alternatives to animal testing in the third revision of the ICHS5 guideline on the detection of toxicity to reproduction for human pharmaceuticals; as stated “A number of alternative in-vitro, ex-vivo, and non-mammalian in-vitro assays (alternative assays) have been developed to detect potential hazards to embryo-fetal development. If properly qualified, alternative assays have the potential to defer or replace (in certain circumstances) conventional in-vivo studies” [2]. The guideline does not specify which in-vitro assays should be used, but, so far, three in-vitro rodent-based toxicity assays (the mouse embryonic stem-cell test, the rodent micromass assay and the rodent whole-embryo culture) have been validated by the ECVAM (the European Centre for the Validation of Alternative Methods). However, another screening model is largely being investigated, yet not validated: the zebrafish (*Danio rerio*) embryo [3,4,5,6,7,8]. In addition to the fact that the zebrafish species has a high reproductive capacity, and that the zebrafish embryo model is not considered an animal model until 120 h post fertilization (hpf) under European regulation (EU Directive 2010/63), it has several advantages compared with the validated in-vitro assays. Zebrafish embryos are a whole-organism model with a translucent chorion that facilitates morphological observation during the entire period of organogenesis. Comparative studies have also shown a high concordance (80–85%) between the findings in zebrafish embryo assays and in-vivo developmental toxicity studies in mammalian models [8,9,10,11]. Despite this fact, a parameter is still under debate for several alternatives to animal testing: the biotransformation capacity of in-vitro models, including the zebrafish embryo [12,13]. Biotransformation is the process by which a xenobiotic compound is biochemically transformed into one or several metabolites to improve its elimination from the body [12]. These first transformations are mainly conducted by Phase I metabolizing enzymes, of which the cytochrome P450 enzymes (CYPs) are the major superfamily involved in the metabolism of both endogenous and exogenous molecules. Regarding drug metabolism, CYP1-4 families are the key players in vertebrates, accounting for the metabolism of approximately 75% of drugs currently on the market [14]. CYPs are membrane-bound enzymes that are present in the endoplasmic reticulum of most cells, although the highest concentrations are found in the liver, followed by the small intestine [15]. The liver and intestine of zebrafish become functional around 96 hpf, coinciding with the end of organogenesis [16,17]. As such, it is questionable whether zebrafish embryos can metabolize and bioactivate xenobiotics during organogenesis, which is the most sensitive period for exposure to developmental toxicants. It is of paramount importance to unravel the biotransformation capacity of this model during organogenesis, as false negatives could occur for compounds that require bioactivation to exert their teratogenic effect, if the metabolic capacity of the zebrafish embryo is too low.

Until now, the metabolic capacity of the zebrafish embryo has mainly been investigated by CYP-non-specific (benzyloxy–methyl–resorufin) [18,19] and -specific (e.g., 7-ethoxyresorufin, for the CYP1 family; 7-benzyloxy-4-trifluoromethylcoumarin-O-debenzylation, for the CYP3A family; 7-methoxycoumarin-O-demethylasem for CYP2 family) [20,21,22,23,24,25,26] fluorescent probes. The activity of these CYP enzymes can also be assessed in the embryos/larvae homogenate microsomes, which are endoplasmic reticulum vesicles containing anchored CYP enzymes on their outer membranes. The amount of CYP enzyme is diluted in embryos/larvae homogenates compared with microsomes from adult zebrafish. In the latter, microsomes are produced solely from organs of interest (e.g., liver, intestine, kidneys) that are often rich in mature CYP enzymes. Recent in-vitro and in-vivo research in zebrafish embryos showed that the mRNA expression of most CYPs—families 1–3—reaches a plateau between 72 and 120 hpf and stays stable throughout larval development (up until 32 days post fertilization). Most importantly, it correlates with the onset of CYP (1–3) activity in developing zebrafish embryos [19]. On the other hand, the transcripts and/or the activity of these Phase I enzymes appeared to be low or undetectable before 72 hpf and only reach mature capacity by 120 hpf. To circumvent the potential lack of metabolization capacity of this model, several groups have tried to co-incubate zebrafish embryos with an exogenous metabolism-activating system (MAS), consisting of adult liver microsomal protein, in the so-called metabolic *Danio rerio* test (mDarT) [27]. However, co-incubation with microsomes causes embryotoxicity in zebrafish embryos, which makes this approach unsuitable for screening purposes [28,29,30]. Moreover, different sources of mammalian adult liver microsomes are currently used for the MAS, such as rat [27,28,29,30,31], which are readily accessible and relatively cheap to generate, and human, which are less accessible and, thus, more expensive when purchased [32,33]. As species differences in drug metabolism are known [32,33,34], this should be taken into account and further investigated to standardize this in-vitro model for human safety and risk assessment. 

A diverse group of compounds, such as mycotoxins (e.g., aflatoxin β1), environmental contaminants (e.g., 2-acetylaminofluorene, benzo[a]pyrene) and drugs (e.g., cyclophosphamide, carbamazepine (CBZ), phenytoin (PHE)), called proteratogens, need to be bioactivated in mammals in order to become teratogenic. The compounds cited have also been shown to induce teratogenicity in zebrafish embryos after exposure from 2.5 to 72 hpf and, hence, concluded as being bioactivated in zebrafish, as in mammals [35]. However, this contrasts with published data on the low-to-absent metabolic capacity of the zebrafish embryo before 72 hpf, as described above, and no measurement of metabolite formation was performed in that study. As such, these compounds may be solely teratogenic in the zebrafish, and do not require any bioactivation of the parent compounds to induce toxicity. Numerous studies have merely focused on gross morphology when exposing zebrafish embryos to xenobiotics for teratogenicity screening. However, the uptake and potential metabolism of the parent compounds should be confirmed, especially when negative results are obtained [36,37,38,39]. Hence, this study aims to further characterize the zebrafish embryo model for developmental toxicity screening of compounds that require bioactivation to exert toxicity. For this purpose, we selected two anti-epileptics, CBZ and PHE, whose active metabolites cause developmental toxicity in mammals. We assessed the morphological effects and internal concentrations of these compounds and their active metabolites, in zebrafish embryos, from 5¼ hpf to 120 hpf. We are focusing on assessing the formation of these active metabolites in zebrafish, as the overall goal is to further characterize this model for human safety. While the zebrafish embryonic stage spans from 0 to 72/96 hpf, the larval stage starts at the end of the embryonic stage and spans until the juvenile stage around 30 days post fertilization. In our study, the exposure window was extended to 120 hpf to investigate the potential formation of the metabolites over time in zebrafish embryos/larvae and observe the induction of additional teratogenic effects by the parent compound and their potential active metabolite(s) beyond 72 hpf. This time point, 72 hpf, appears to be a threshold for CYPs activity in zebrafish embryos [19]. In addition, in vitro metabolism of these compounds in adult zebrafish was also compared with human and other routinely used species in the in-vivo developmental toxicity studies, the rat, and the rabbit, to get a better view of potential species differences. The main mammalian active metabolites for CBZ and PHE are carbamazepine-10,11-epoxide (E-CBZ) and 5-(4-hydroxyphenyl)-5-phenylhydantoin (HPPH), respectively, which are known to be responsible of developmental toxicity in human. CBZ is metabolized to its main active metabolite by CYP3A4 and CYP2C8 in humans [40,41], CYP2B1/CYP2B2 and CYP3A isoforms in rats [42,43] and CYP3A6 isoform in rabbits [44]. For PHE, the mammalian toxic metabolite HPPH is formed by the isoforms CYP2C9 and CYP2C19 in humans [45,46,47], CYP3A1/CYP3A2 isoforms in rats [48] and CYP2C3 in the rabbit [49,50]. For embryonic, larval, juvenile and adult zebrafish, it still is unknown whether these compounds are metabolized and, if so, whether the same active metabolites as in mammals are formed and cause developmental toxicity. Our study has focused on the zebrafish embryo/early larva model and the characterization of the metabolic pathways of compounds that need to be bioactivated to exert their toxicity in human, such as CBZ and PHE. Therefore, we explored whether zebrafish embryos/early larvae (as well as adult fish) biotransform CBZ and PHE to metabolites known to be teratogenic in humans and whether these metabolites are indeed involved in the teratogenic effect in zebrafish.

Morphological abnormalities and developmental delays were observed after incubating the two anti-epileptics drugs, CBZ and PHE, with zebrafish embryos during organogenesis. However, these effects were not observed after direct exposure to E-CBZ and HPPH. While whole zebrafish larvae formed E-CBZ after exposure to CBZ, surprisingly, HPPH was not formed after exposure to PHE. At the adult stage, our in-vitro investigation with CBZ and PHE in human and the non-clinical species (rat and rabbit) liver microsomes demonstrated the formation of E-CBZ and HPPH. However, sharp distinct turnover rates were observed after 240 min of exposure between the species investigated and the zebrafish. Therefore, the findings of this study emphasize the need to develop evidence-based strategies when using the zebrafish embryo for developmental toxicity screening, including uptake assessment of the compounds and potential metabolite formation in parallel to gross morphological investigations.

## 2. Results

### 2.1. Exposure of Zebrafish Embryos and Larvae to CBZ and PHE—Gross Morphological Investigation

#### 2.1.1. CBZ and Its Metabolite E-CBZ 

**Figure 1:**
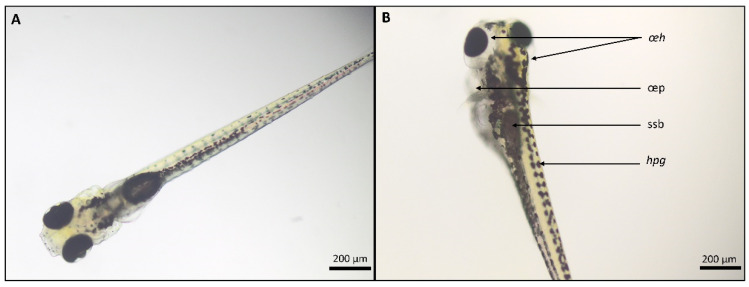
Larvae at approximately 115 h post fertilization (hpf). (**A**): Larva showing no signs of abnormalities after exposure to EMS + 0.5% DMSO. (**B**): Larvae with abnormalities after exposure to 250 µM CBZ (59 µg/mL) in EMS + 0.5% DMSO. oeh: edema head; oep: edema pericard; ssb: small swim bladder; hpg: high pigmentation. Bar scale: 200 µm.

All experiments were valid, since less or equal to 10% of coagulated and or malformed embryos were observed in all control groups (embryo medium solution (EMS); EMS + 0.5% DMSO and EMS + 1% DMSO). Each replicate (*n* = 2) consisting of 20 embryos were statistically and independently analyzed. No statistically significant differences were observed before 48 hpf (Supplementary Figure S1A, see Supplementary Materials) in any of the CBZ concentrations (31.25 µM; 85 µM, 250 µM) and the E-CBZ concentration (250 µM) compared with the controls. Starting from 48 hpf, we observed a statistically significant delay in hatching in the second biological replicate (*p* = 0.0002) for the 85-µM CBZ test group (see Supplementary Figure S1B). This delay in development at 85 µM CBZ was recovered at the later time points (72, 96 and 120 hpf). A hatching delay was also observed at the highest CBZ concentration, 250 µM, at 72 hpf for both replicates (*p* = 0.0083, *p* = 0.0033; see Supplementary Figure S1C), and remained present throughout 96 hpf (*p* = 0.0471; see Supplementary Figure S1C) and 120 hpf (*p* = 0.0471; see Table 1) for the first replicate, demonstrating variation within replicates at 96 and 120 hpf. A delay of swim bladder development and inflation in the first replicate was also noticeable for the highest CBZ concentration, 250 µM, starting at 72 hpf (*p* = 0.031; see Supplementary Figure S1C) and persisted until 96 hpf (*p* = 0.0095; see Supplementary Figure S1D). At 120 hpf, a defect of swim bladder inflation was observed for both replicates (*p* ≤ 0.0001, *p* = 0.0057) in the 250-µM CBZ group (Figure 1), but also for one replicate of the 31.25 µM CBZ (*p* = 0.0033) and 85-µM CBZ (*p* = 0.0083) concentrations. Therefore, a delay in swim bladder inflation appeared to be a fingerprint of CBZ exposure to zebrafish embryos and larvae in our investigation. Moreover, starting from 96 hpf and throughout 120 hpf, abnormal pigmentation was observed in the 250-µM CBZ group. For the two final time points, darker and wider pigmentation dots were observed compared with the 0.5%-DMSO control in both replicates (*p* ≤ 0.0001 for both; Figure 1 and Table 1). At 120 hpf, a slight increase in pericardial edemas was also observed at 250 µM CBZ in the first replicate (*p* = 0.0471). In contrast, at the same time point (120 hpf) a strong presence of head and eye edemas was observed in both replicates (*p* = 0.0471, *p* = 0.0197) of the 250-µM CBZ group, characterized by the formation of translucid tissues around the eyes and a bump on the top of the head (Figure 1 and Table 1). The only significant effect observed after exposure of the embryos and larvae to the metabolite E-CBZ (250 µM) was a delay in swim bladder inflation at 120 hpf for one replicate (Table 1).

#### 2.1.2. Phenytoin and Its Metabolite HPPH

**Figure 2:**
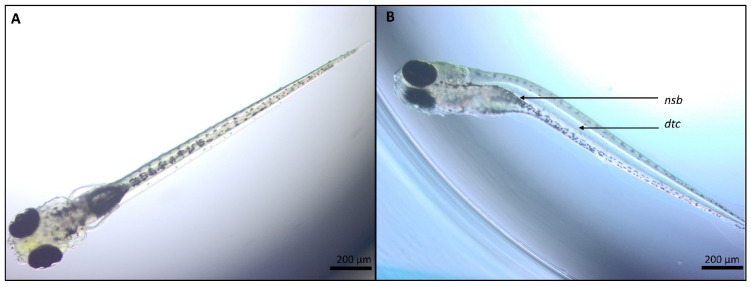
Larvae at approximately 115 h post fertilization (hpf). (**A**): Larva showing no signs of abnormalities after exposure to EMS + 0.5% DMSO. (**B**): Larva with abnormalities after exposure to 250 µM PHE (63 µg/mL) in EMS + 0.5% DMSO. nsb: no swim bladder inflation; dtc: downward tail curved. Bar scale: 200 µm.

All experiments were valid, as less or equal to 10% of coagulated and or malformed embryos were observed in all control groups (EMS; EMS + 0.5% DMSO). Each replicate (*n* = 2) consisting of 20 embryos were statistically and independently analyzed. No statistically significant differences were found in the control groups at any of the investigated time points or within replicates. The first observed statistically significant difference between the test groups was at 48 hpf (See Supplementary Figure S2A), at which a delay of embryonic hatching was noticeable at 250 µM PHE for both replicates (*p* ≤ 0.0001, *p* = 0.0095) compared with the solvent control group (EMS + 0.5% DMSO). However, this delay was no longer present at 72 hpf. The solvent controls showed a statistically significant difference for the swim bladder inflation at 250 µM PHE for the second replicate at 72 hpf (*p* = 0.0031; see Supplementary Figure S2B), for both replicates at 96 hpf (*p* ≤ 0.0001; see Supplementary Figure S2C) and one replicate at 120 hpf (*p* ≤ 0.0001; Figure 2 and Table 2). An appreciable delay in swim bladder elongation was observed at 31.25 µM PHE for one of the replicates at 96 hpf (*p* = 0.0084; see Supplementary Figure S2B) and for 85 µM PHE (*p* = 0.0004; see Supplementary Figure S2C). For the last time point, 120 hpf, the delay was no longer observed in the 31.25-µM PHE test group but remained present in the second replicate at 85 µM PHE (*p* = 0.0008; see Table 2). We therefore see a clear effect on the swim bladder inflation for PHE but with variations within replicates. Additional malformations were observed at 250 µM PHE, including lower pigmentation starting from 72 hpf (*p* = 0.0471; see Supplementary Figure S2B) until 96 hpf (*p* = 0.0084; see Supplementary Figure S2C) and at 120 hpf (*p* = 0.0436; see Supplementary Figure S2C) for one replicate. Lower pigmentation was observed for the 31.25-µM and 85-µM PHE concentrations for some larvae at 120 hpf, albeit, not to a statistically different level (Table 2). The last noticeable abnormality at 250 µM PHE was an inward curvature of the larval tails at 120 hpf for the first replicate (*p* = 0.0083; Figure 2 and Table 2). Therefore, the sole fingerprint observable after exposure of zebrafish embryos and larvae to PHE was a delay in swim bladder inflation. Another compelling effect was a delay in embryonic hatching at 48 hpf, as it was noticeable in both replicates for 250 µM PHE. Strikingly, no significant effect was observed on zebrafish embryos/larvae development after exposure to 250 µM HPPH (Table 2).

### 2.2. Exposure of Zebrafish Embryos and Larvae to CBZ and PHE—Analytical Investigation

#### 2.2.1. Quantification of CBZ and PHE, and Their Metabolites E-CBZ and HPPH, in Medium Samples after Exposure

For the first drug CBZ, the quantified concentration in the stock and culture medium for the lowest concentration, 31.25-µM CBZ, matched the nominal concentration and remained stable over time (Table 3). For the medium (85 µM) and highest (250 µM) CBZ concentrations, the quantified concentrations of the stock solutions were between 10 to 20% lower than the nominal concentrations at the two time points (0 and 120 hpf). In contrast, quantified concentrations were 10 to 20% higher than the nominal concentrations in the culture medium samples exposed to embryos/larvae at 48, 96 and 120 hpf. The difference between the quantified concentrations in the stock and culture medium was mostly pronounced for the 85-µM concentration. For the quantified concentration of E-CBZ in the culture medium at 96 hpf, the quantified concentration was as for CBZ, around 20% above the nominal concentration (Table 3). Therefore, we could not observe a decrease of CBZ in the culture medium samples but, rather, stable CBZ concentrations over time for the three concentrations investigated. No potential metabolism of the parent compound, CBZ, could therefore be concluded from the culture medium investigation.

For our second drug PHE, the quantified concentration in culture medium for the lowest concentration, 31.25 µM PHE, remained stable over time and were 20% lower than the nominal concentration, which was higher than the concentration in the stock medium (Table 4). For the medium concentration, 85 µM PHE, quantified concentrations of the stock medium at 0 hpf and culture medium at 48 hpf were around 20% lower than the expected concentration. In contrast to the first time points, a decrease in both the culture medium at 96 and 120 hpf and the stock medium at 120 hpf was observed for the 85-µM PHE group, as the concentrations quantified were around 35% lower than the nominal concentrations. As we also observed a drop in the quantified concentration in the 85-µM stock solution at 120 hpf, we suspected that PHE precipitated in EMS. For the 250-µM PHE test group, all concentrations in the stock and culture medium at all the different time points did not reach more than 45% of the nominal concentrations (Table 4), so 55% lower than expected. As the quantified concentrations decreased in a concentration-dependent manner, it indicated that PHE did not dissolve properly in the EMS and/or precipitated in EMS as visually observed in the stock medium for 85 and 250 µM PHE. Therefore, embryos and larvae were not exposed to the expected concentration. Moreover, as for CBZ, no clear indication of PHE metabolization was observed in the mediums as quantified concentrations remained stable over time except for the 85-µM PHE group where compound precipitation was observed over time. The quantified concentrations for HPPH reached at 96 hpf around 90% of the nominal concentration and was therefore better diluted in the EMS in contrast to its analogue parent compound, PHE.

#### 2.2.2. Quantification of the Metabolites E-CBZ and HPPH in the Culture Medium after Exposure to the Parent Compounds 

**Figure 3:**
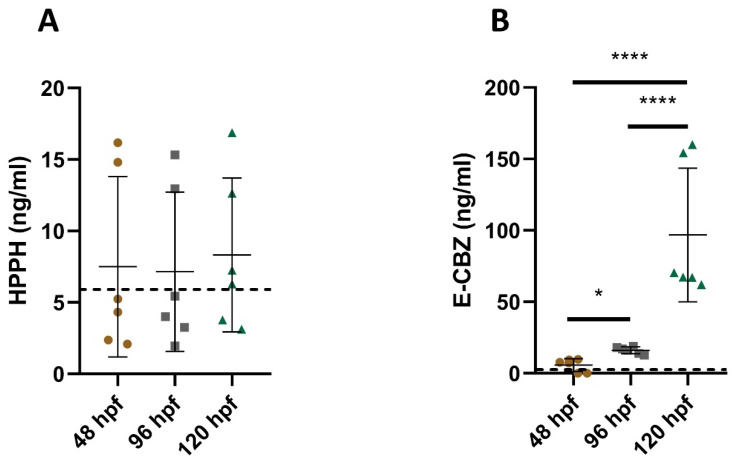
Quantified HPPH (**A**) and E-CBZ (**B**) in the pooled culture medium exposed to zebrafish embryos/larvae—PHE 250 µM (63 µg/mL) and CBZ 250 µM (59 µg/mL). Each biological replicate was performed in technical duplicates, all represented in the graphs. The bold dashed line (- - -) represents the LLOQ of the compound investigated. Hpf: hours post fertilization; * *p* < 0.05, **** *p* ≤ 0.0001.

Regarding the potential formation of metabolites in culture medium, no metabolites were detected at the lowest (31.25 µM) and medium concentrations (85 µM) for both anti-epileptic drugs (data not shown). Overall, for all time points investigated after the exposure of embryos/larvae to 250 µM PHE, only very low to unquantifiable HPPH concentrations were observed, with large standard deviations. The average quantified concentrations were 7.5 ± 6.3 ng/mL at 48 hpf, 7.2 ± 5.6 ng/mL at 96 hpf and 8.3 ± 5.4 ng/mL at 120 hpf (Figure 3A). Therefore, no clear formation and increase over time of the metabolite HPPH can be observed in our 250-µM PHE pooled culture medium. In contrast, for the 250-µM CBZ culture medium, E-CBZ formation could be detected at a very low concentration, as early as 48 hpf in two out of the three replicates processed (9.5 ng/mL and 7.5 ng/mL). Furthermore, the average quantified E-CBZ concentration in the pooled culture medium slowly increased in the 96 hpf samples (16.2 ± 2.4 ng/mL) and 120 hpf (96.9 ± 46.8 ng/mL) (Figure 3B). The metabolism of CBZ into E-CBZ seemed to soar upward with a six-fold factor between 96 and 120 hpf with only 24 h of exposure to the larvae. The quantified concentrations at 120 hpf were distinct to those quantified at 48 and 96 hpf (*p* ≤ 0.0001 for both).

#### 2.2.3. Quantification of the Parent Compounds CBZ and PHE, and Their Metabolites E-CBZ and HPPH, in Zebrafish Embryos/Larvae Homogenate Extracts

##### Quantification of CBZ and PHE, and Their Metabolites E-CBZ and HPPH, in Zebrafish Embryos/Larvae Homogenate Extracts after Direct Exposure

**Figure 4:**
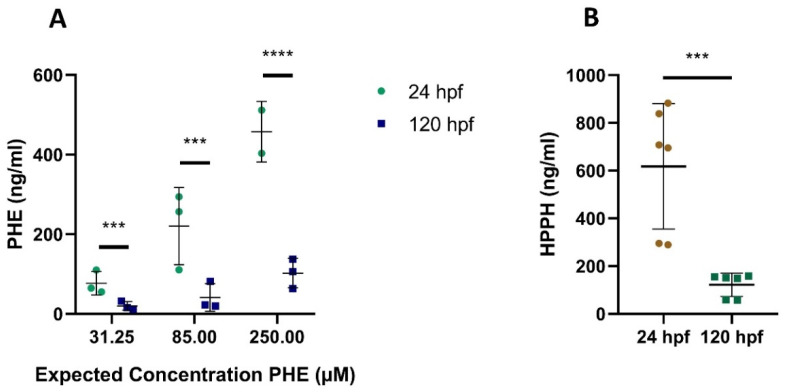
Quantified PHE and HPPH in extraction samples from whole zebrafish embryos/larvae at 24 and 120 h post fertilization (hpf) after exposure to PHE 31.25 µM (7.88 µg/mL), 85 µM (21.4 µg/mL), 250 µM (63 µg/mL) (**A**) and HPPH 250 µM (67 µg/mL) (**B**). For (**A**) each value represents the average of the technical duplicates, whereas each technical duplicate is presented in (**B**). *** *p* ≤ 0.001; **** *p* ≤ 0.0001.

**Figure 5:**
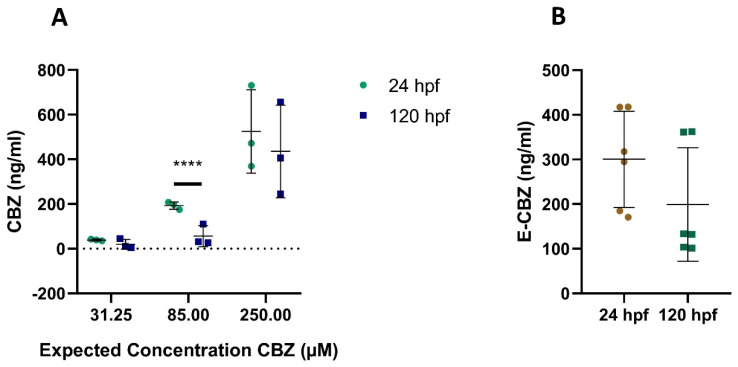
Quantified CBZ and E-CBZ in extraction samples from whole zebrafish embryos/larvae at 24 and 120 h post fertilization (hpf) after exposure to CBZ 31.25 µM (7.38 µg/mL), 85 µM (20 µg/mL), 250 µM (59 µg/mL) (**A**) and to E-CBZ 250 µM (63 µg/mL) (**B**). For (**A**) each value represents the average of the technical duplicates, whereas each technical duplicate is presented in (**B**). **** *p* ≤ 0.0001.

For the three concentrations of CBZ and PHE, we had clear indication that the compounds were taken up by the embryos and larvae at the two sampled time points, 24 and 120 hpf (Figure 4A and Figure 5A) as quantification of the compounds in the homogenate extracts was achieved. Uptake of the compounds was also observed after direct exposure of zebrafish embryos/larvae to the metabolites E-CBZ and HPPH (Figure 4B and Figure 5B). The four compounds did not bioaccumulate in embryos/larvae over time. On the contrary, all concentrations of PHE and HPPH were significantly lower at 120 hpf when compared with 24 hpf (Figure 4A,B), whereas for the other anti-epileptic drug, CBZ, this was only significant at 85-µM CBZ (3.4-fold lower at 120 hpf than at 24 hpf) (Figure 5). The ratio differences between the two time points was higher (approximatively five-fold) for PHE and HPPH than for CBZ and E-CBZ (Figure 4 and Figure 5).

##### Quantification of the Metabolite, E-CBZ and HPPH, in Embryos and Larvae after Exposure to CBZ and PHE

**Figure 6:**
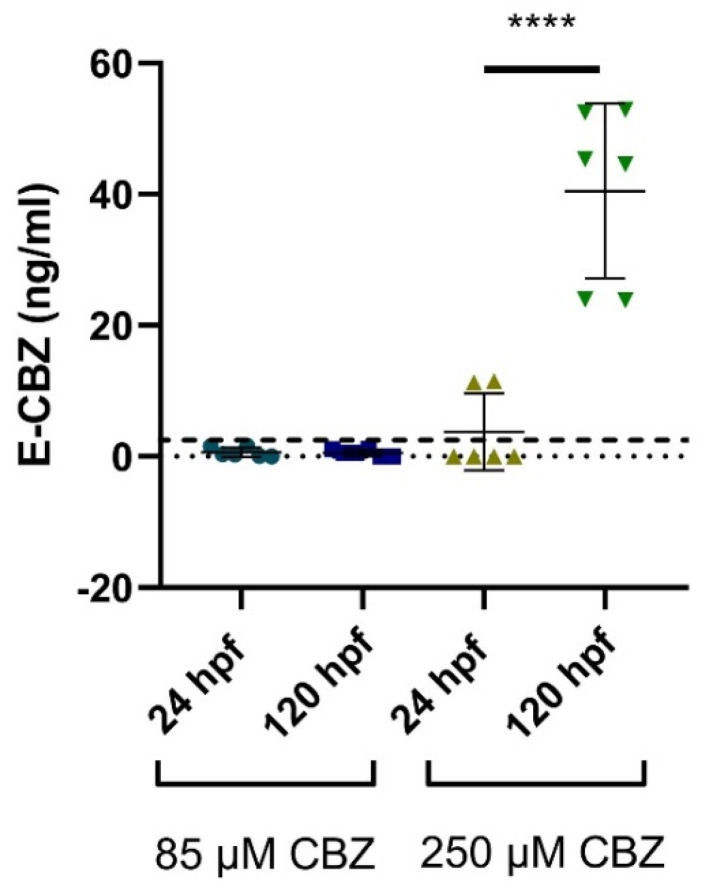
Quantified E-CBZ in extraction samples from whole zebrafish embryos/larvae at 24 and 120 h post fertilization (hpf) after zebrafish embryo exposure to CBZ 85 µM (20 µg/mL)–250 µM (59 µg/mL). Each biological replicate was performed in technical duplicate, all represented in the graph. The bold dashed line (- - -) represent the LLOQ of the compound investigated. **** *p* ≤ 0.0001.

We could not detect the presence of HPPH in any samples after exposure to PHE, at the two different time points, 24 hpf and 120 hpf. For the embryos/larvae exposed to CBZ, E-CBZ could not be detected at the lowest concentration of CBZ (31.25 µM) at 24 and 120 hpf. For the mid concentration of CBZ (85 µM), E-CBZ could be detected at the two time points, although, at concentrations below the LLOQ (1.5 ng/mL) (Figure 6). For the highest CBZ concentration, 250 µM, E-CBZ was detected at 24 hpf (average concentration 11.38 ± 0.1 ng/mL), but only in one out of the three technical replicates (Figure 6). At 120 hpf, an average concentration of 40.5 ± 13.3 ng/mL E-CBZ was detected and well-quantified. The average E-CBZ quantified concentration was significantly different between the two time points (*p* ≤ 0.0001).

### 2.3. In-Vitro Metabolism of CBZ and PHE: A Cross-Species Comparison

During a pilot investigation focusing on the parent compounds, only a slight decrease of CBZ was detected at 240 min after exposure to rabbit adult microsomes and no changes were observed for the other species. Similarly, only a very slight decrease in PHE concentration was observed after exposure of 240 min to rat and rabbit adult liver microsomes. No decrease of PHE was observable after exposure to zebrafish and human adult liver microsomes and to supersomes (data not shown). As no clear consumption of the parent compounds was observed, we therefore focused on the potential formation of the mammalian active metabolites in our samples. 

#### 2.3.1. Quantification of E-CBZ after Microsomal Incubation with CBZ

**Figure 7:**
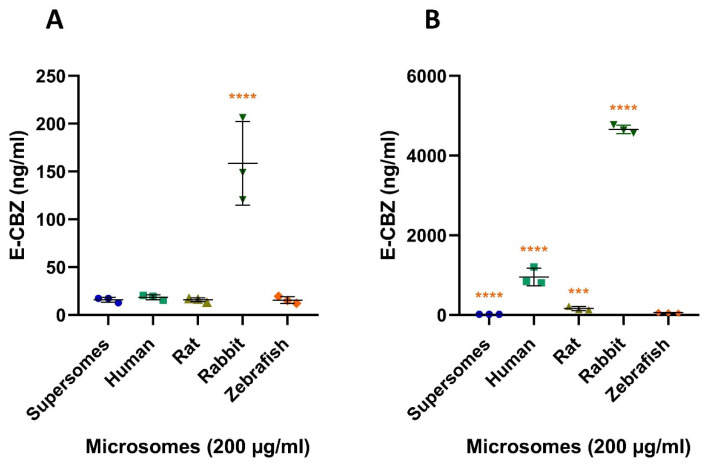
E-CBZ formation after 5 (**A**) and 240 min (**B**) exposure of supersome (negative control), human microsomes (positive control), rat, rabbit and zebrafish microsomes (200 µg/mL) to 100 µM (23.62 µg/mL) CBZ. Each microsomal result is compared with adult zebrafish liver microsomes reaction. Each point represents the mean of two technical replicates. *** *p* ≤ 0.001, **** *p* ≤ 0.0001.

We quantified an average concentration of 15.9 ± 2.7 ng/mL (Figure 7A) E-CBZ in supersomes reaction mix after five minutes of incubation, and 16.6 ± 1.8 ng/mL (Figure 7B) after 240 min. As supersomes were our negative control and no variation was detected between 5 and 240 min, the E-CBZ concentrations quantified at the two time points represented E-CBZ contamination in the reaction mixture. We, thus, concluded that only contamination of E-CBZ was present at 5 min for the reaction mix with human (18.5 ± 2.6 ng/mL), rat (15.9 ± 2.1 ng/mL) and zebrafish microsomes (15.6 ± 3.7 ng/mL). In contrast to the other species, CBZ was already metabolized in E-CBZ by the adult rabbit liver microsomes at 5 min with an average quantified concentration of 158.7 ± 43.7 ng/mL and this was statistically different from the zebrafish (*p* ≤ 0.0001). For human microsomes, a clear increase of E-CBZ was observed with a quantified average concentration of 953.8 ± 222.2 ng/mL at 240 min. For the rat, rabbit, and zebrafish, the quantified average E-CBZ concentrations were 162.2 ± 48.2 ng/mL, 4658.1 ± 104.3 ng/mL and 56.3 ± 7.7 ng/mL, respectively (Figure 7B), after 240 min of incubation. The average concentration quantified in the zebrafish liver microsomal reaction was significantly different from the concentration quantified in the samples with rabbit and human liver microsomes (*p* ≤ 0.0001 for both, Figure 7B) and to the rat (*p* = 0.0001). The turnover of CBZ to E-CBZ in zebrafish liver microsomes was 0.24%, which is significantly lower than for rat (0.69%), human (4.03%) and rabbit (19.72%). The turnover of the parent compound to the metabolite was calculated as the percentage of quantified metabolite concentration formed versus the initial concentration of the parent compound. 

#### 2.3.2. Quantification of HPPH after Microsomal Incubation with PHE

**Figure 8:**
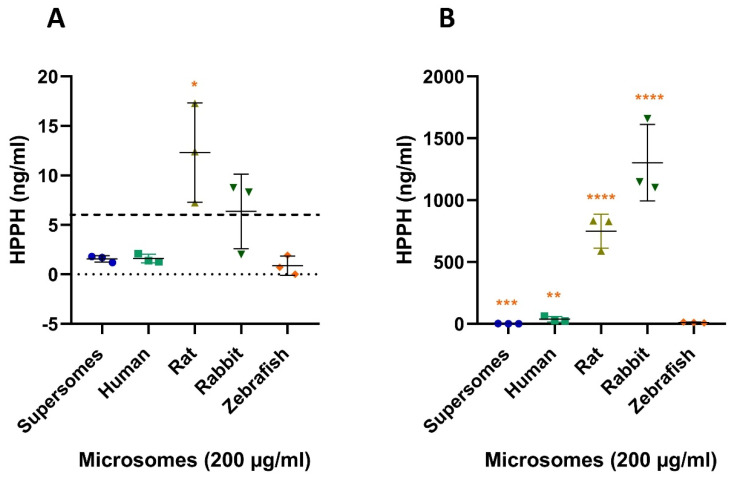
HPPH formation after 5 (**A**) and 240 min (**B**) exposure of supersomes (negative control), human microsomes (positive control), rat, rabbit and zebrafish microsomes (200 µg/mL) to 100 µM (25.23 µg/mL) PHE. Each microsomal result is compared with adult zebrafish liver microsomes reaction. The bold dashed line (- - -) represent the LLOQ of the compound investigated. Each point represents the mean of two technical replicates. * *p* ≤ 0.05, ** *p* ≤ 0.01, *** *p* ≤ 0.001, **** *p* ≤ 0.0001.

For PHE, traces of HPPH were also detected in our negative control but at concentrations below the LLOQ (6 ng/mL) at 5 and 240 min. The same was observed for our human and zebrafish adult liver microsomal reactions at 5 min. In contrast, for the rat and the rabbit, concentrations just above the LLOQ were detected, with concentrations of 12.3 ± 5.0 ng/mL for rat and 6.4 ± 3.8 ng/mL for rabbit (Figure 8A) at 5 min. After 240 min of incubation, only a slight increase of HPPH formation was observed for zebrafish adult liver microsomes, with an average quantified concentration just above the LLOQ, 10.4 ± 3.7 ng/L. For the rat liver microsomes, an average HPPH concentration of 749.3 ± 138.6 ng/mL was quantified and 1303.0 ± 308.9 ng/mL for the rabbit liver microsomes (Figure 8A). Hence, the average concentration quantified in zebrafish liver microsomal reaction is different from the concentration quantified in the samples with rat and rabbit liver microsomes (*p* ≤ 0.0001 for both, Figure 8B). For our positive control with human liver microsomes, the concentration quantified (36.9 ± 21.9 ng/mL) was still statistically different (*p* ≤ 0.001) to zebrafish but to a lower extent than rat and rabbit. The turnover of PHE to HPPH in the zebrafish reaction mixture was very low (0.06% after 240 min of incubation). However, it is the closest species to the turnover observed for human (0.15%) as we observed much higher turnovers for rat (2.97%) and rabbit (5.16%) liver microsomes.

## 3. Discussion

This study had two objectives. First, to assess the morphological outcomes and potential bioactivation of two anti-epileptic drugs, CBZ and PHE, in zebrafish embryos during organogenesis from 5¼ hpf to 120 hpf. Second, to investigate potential species differences in CBZ and PHE metabolism with zebrafish, rat, rabbit and human liver microsomes.

For the gross morphology investigation, zebrafish embryos/larvae were exposed to the lowest (31.25 µM) and highest (250 µM) concentrations of CBZ and PHE selected by Weigt et al. [35], including a mid-concentration of 85 µM. As the aim was to assess the morphological effects of the parent compounds and their active metabolites in zebrafish during the entire period of organogenesis, we extended the exposure period from 72 hpf (as in Weigt et al. [35]) up to the free-feeding stage (120 hpf). Most findings of our morphological investigation were in accordance with Weigt et al. Our investigation demonstrated that embryos/larvae were mostly affected in the 250-µM test groups, and the first effects could be observed from 48 hpf onwards for both compounds. Growth retardation was observed with a significant delay in hatching at 48 hpf and 72 hpf for 250 µM PHE but for CBZ it was only observable at 72 hpf. Those results are also in accordance with other studies for PHE [51,52], but no developmental delays were reported yet for CBZ, to our knowledge. However, differences for some morphological endpoints were also observed from the investigation of Weigt et al. [35]. For PHE, only a downward curvature of the tail at 120 hpf in the 250-µM PHE group for one replicate was detected, whereas Weigt et al. observed this as early as at 72 hpf. The upward tail curvature at 72 hpf for CBZ was also not observed in this study. A potential explanation for this discrepancy, could be the fact that Weigt et al. dechorionated larvae that had not hatched by 72 hpf. All dechorionated embryos showed the upward bending tail malformation and were scored six hours later for tail stretching. In our study, the larvae were not dechorionated at 72 hpf and only observed again 24 h later. The curvature of the tails observed by Weigt et al. could be a transient effect linked to the delay in hatching induced by the 250-µM CBZ exposure concentration. Therefore, extending the exposure window could help to distinguish transient from teratogenic effects. Moreover, it could also provide us additional information on the organ/functions that could be affected by the exposure of the compound and/or its active metabolite(s), at a later stage of organogenesis. For CBZ and PHE, many induced teratogenic effects were unraveled starting from 72 hpf. The additional data could provide key information for the development of their corresponding adverse outcome pathways (AOP). For both compounds, developmental delay seems to be persistent at 120 hpf, as a delay in swim bladder development and inflation was observed at 96 and 120 hpf. PHE and CBZ also had an impact on pigmentation at their highest concentrations. Less pigmentation was observed at 250 µM PHE, in both replicates, at 72 and 96 hpf and, in one replicate, at 120 hpf. In contrast, a stronger pigmentation with enlarged pigmentation dots was observed at 96 and 120 hpf in larvae exposed to 250 µM CBZ. Pericardial edema was also observed at the last time points in the CBZ 250-µM group, which was already reported at 48–72 hpf in other studies [53,54,55]. Starting from 96 and 120 hpf, we observed the formation of head/eye edemas in the CBZ 250-µM group, which had not been yet reported. A study from Pohl et al. [56] demonstrated that exposure of zebrafish embryos to CBZ was linked to the downregulation of *dct* and *tyr* genes’ expressions. While dct (Dopachrome tautomerase) is a melanogenic enzyme and is an early marker for all melanin-synthesizing cells, both in neural crest-derived and in the pigmented retinal epithelium [57], tyr (Tyrosinase) is implicated in the formation of retinal pigmented epithelium, melanin biosynthesis and melanophores and iridophores stripe patterning in zebrafish. However, in this study, the authors have concluded that even if those genes were downregulated after CBZ exposure, CBZ alone, could not cause transcriptional effects. However, exposure to ozonated CBZ, and therefore CBZ by-products, downregulated a network of genes (*dct*, *tyr*, *pmelb* (Premelanosome protein), *cax1* (Vacuolar cation/proton exchanger 1), *mlana* (Melanoma antigen recognized by T cells 1), *slc24a5* (Solute carrier family 24 member 5), *syngr1a* (Synaptogyrin-1a), *tnn* (Tenascin N) and *zgc:91968*) that could have a transcriptional effect on pigmentation, neural crest cells and swim bladder inflation. They also observed that the expression of *dct* and *tyr* in the ozonated CBZ group was significantly lower than the group solely exposed to CBZ and seems to indicate that ozonation, and therefore CBZ by-products, exacerbates the repressive effects of CBZ on those specific genes. The latter suggests that several teratogenic effects induced by CBZ at 96 and 120 hpf in our study might be due to the presence of CBZ metabolite(s). While for CBZ, the percentage of larvae affected at 72 hpf in our study were for both concentrations in accordance with Weigt et al. [35] (10.0 ± 5.0% for 31.25-µM CBZ and 48.3 ± 11.5% for 250 µM CBZ at 72 hpf), the percentage of larvae affected by PHE in our study was 20% lower for both concentrations (18.3 ± 7.6% for 31.25 µM PHE and 45.0 ± 21.8% for 250 µM). More importantly, the effects of the metabolites HPPH and E-CBZ on zebrafish embryo development were absent or very mild, suggesting that PHE and CBZ might not require bioactivation to exert their teratogenic action in zebrafish embryos or that other metabolites than HPPH and E-CBZ are formed and teratogenic in zebrafish. While direct exposure to 250 µM HPPH did not affect the normal development of zebrafish embryos/larvae during the whole period of investigation, 250 µM E-CBZ only reduced the rate of swim bladder inflation at 120 hpf. A lack of findings was also reported after the incubation of zebrafish embryos from 3 to 144 hpf to a lower concentration of E-CBZ, 2.6 mg/L (10.3 µM), in a study from Pohl et al. [58]. For another so-called human proteratogen, trimethadione (TMO), we reported earlier that the effects of the parent compound, TMO, were stronger than the direct exposure to the active metabolite dimethadione (DMO) on zebrafish development [29]. In this study, it was demonstrated that both compounds induced teratogenicity after 70 h of exposure (time point 72 hpf). Thus, the bioactivation of TMO into DMO is not essential to exert teratogenicity for zebrafish. 

As differences in morphological outcome between species may be due to differences in the uptake of compounds, we investigated this further. No clear, or very low, consumption of CBZ and PHE could be observed over time in the culture medium to which the zebrafish embryos/larvae were exposed. On the contrary, higher concentrations (up to 20%) than the expected nominal concentrations were noted in the culture medium. Brandhof and Monforts [54] reported similar findings, in which the average quantified CBZ concentration was 22.3% higher than the nominal concentration after 70.5 h of exposure (time point 72 hpf). A study investigating the potential evaporation of culture medium in a 96-well plate, with the lid in place and incubated at 28.0 ± 0.5 °C, found that 9.46% of the buffer had evaporated at 96 hpf [59]. The evaporation of the culture medium could, then, explain the over-quantification of the compounds in the exposed medium of our study. Therefore, medium change seems important not only to avoid medium acidification and oxygen deprivation, but also to continuously match the targeted concentration over time. For PHE, precipitation in the culture medium was visually noted at 85 and 250 µM, which also occurred in Weigt et al. [35]. The latter resulted in lower exposure concentrations (on average, 65% to 55%, respectively) than the nominal concentrations. As more variability was observed in the PHE quantification compared with CBZ, the solubility issues with PHE may also explain the higher variability in morphological outcome within replicates of our study and with other reported studies. Moreover, it could also explain the lack of studies investigating this anti-epileptic drug with the zebrafish embryo model. Still, HPPH could not be detected in any of the pooled PHE culture medium for all the time points investigated. So, we can safely state that no formation of the metabolite, HPPH, occurred over time for this compound, or at least to a non-biologically relevant concentration. In contrast, a clear formation of E-CBZ was detected at 96 and 120 hpf, indicating biotransformation of CBZ, even when no consumption could be observed of the parent compound in the 250-µM CBZ pooled medium over time.

Investigating the uptake of compounds by zebrafish embryos is important, as it should only occur via diffusion and highly dependent on the physicochemical properties of the compounds. Uptake of the four compounds by the zebrafish embryos and larvae was confirmed in the 24 and 120 hpf whole-body extracts. As E-CBZ and HPPH were taken up by the embryos and larvae, but no teratogenic effect was observed during gross morphology investigation, we can safely conclude that those metabolites are not teratogenic in zebrafish, in contrast to that reported for mammals. Additionally, bioaccumulation of the four compounds in the larvae over time did not occur. For all concentrations of CBZ and E-CBZ, the quantified concentrations were 1.2 to 2 times higher at 24 hpf than at 120 hpf, and even 3.4 times higher for 85 µM CBZ. For PHE and HPPH, all the quantified concentrations were, on average, 5 times higher at 24 hpf than at 120 hpf. This decrease in internal concentrations over time could be indicative of direct elimination of the parent compounds or biotransformation, as also suggested by others [39], to more hydrophillic metabolites that can then be excreted by drug transporters, such as the ATP-binding cassette (ABC), Abcb4. We have already demonstrated that Abcb4 mRNA is expressed during organogenesis and that its expression gradually increases, in developing embryos, from 24 to 120 hpf [19]. For CBZ, this is plausible, as a clear formation of E-CBZ was noticeable in 120 hpf pooled larval extracts compared with 24 hpf extracts, with an average concentration of 40.5 ± 13.3 ng/mL. The results of our larval extracts correlate well with the results of the 250-µM CBZ culture medium samples at 120 hpf, in which E-CBZ was detected at 96 and 120 hpf. As such, the same metabolic pathway as found in mammals seems to be present in zebrafish embryos and larvae for CBZ. However, biotransformation is not always the reason for the observed differences in internal concentrations over time. For PHE, HPPH was not detected in any of the zebrafish embryos/larvae extract samples. Those results are in accordance with the absence of HPPH in the pooled PHE 250 µM culture medium samples. Therefore, zebrafish embryos/larvae do not seem to biotransform PHE to HPPH, in contrast to mammals [50,60,61]. The higher internal concentrations of PHE and HPPH at 24 hpf than at 120 hpf must consequently have other causes. The chorion could play a role here, as we chose not to dechorionate embryos at 24 hpf in our study (see Section 4.2.1), and, more specifically, compound sticking to the chorionic membrane, even after several rinsing steps, could be a reasonable cause of the higher internal quantification at 24 hpf. For a large list of other compounds, an average overestimation of the internal concentration of 5–30 ng/organism has already been reported for embryos with a chorion compared with dechorionated embryos [62]. Another explanation could be the bioaccumulation of compounds in the yolk sac, which is only fully resorbed at 160 hpf. At 26 hpf, the yolk composes around 80% of the dry weight of the whole embryo and is rich in phospholipoproteins. Halbach et al. [63] demonstrated, for several compounds, including CBZ, an overestimation of the internal concentration in the embryonic body due to accumulation in the yolk sac, up to a factor of five after 24 h of exposure [63]. The authors also suggested that, for substances with higher hydrophobicity, such as PHE, it takes longer to reach a steady-state concentration between the yolk and the embryonic body. As such, this could also contribute to our findings for PHE. This reiterates the importance of the physicochemical properties of a compound for its uptake and quantification in whole-body homogenates. 

So, our morphological and analytical assessments suggest that CBZ and PHE are teratogens, and do not require bioactivation in the zebrafish, although we cannot rule out potential roles of other metabolites, aside from HPPH and E-CBZ. Halbach et al. [63] detected the metabolites acridine and 3-OH-carbamazepine in the pmol/embryo range in 96-hpf embryos after exposure to CBZ with an untargeted ultra-high performance liquid chromatography-quadrupole time-of-flight mass spectrometry (UHPLC/Q-TOF-MS) method. Pohl et al. [64] also detected another metabolite, 10,11-dihydrocarbamazepine, in zebrafish embryo culture after exposure to CBZ. Therefore, E-CBZ might not be the sole metabolite formed after CBZ exposure in zebrafish larvae. Further untargeted UHPLC-QTOF-MS investigations, as already performed for a variety of compounds exposed to zebrafish embryos [38,39,63,65,66], could aid in further understanding of the teratogenicity of CBZ and PHE. In this study, a targeted search was only performed for E-CBZ and HPPH, which are known to be teratogenic in mammals, with a targeted UPLC-QqQ-MS method. Roles for active metabolites are plausible for CBZ-induced teratogenicity in zebrafish embryos, as teratogenic effects have already been shown to be much higher after exposing zebrafish embryos to the by-products formed after exposing CBZ to electrochemistry [55] than to CBZ itself. This study, by Zhu et al., suggests that acridine, a by-product of CBZ after its exposure to electrochemistry, was the main toxic by-product out of seven examined. As acridine was also found in the study from Halbach et al. [63] after direct incubation of zebrafish embryo to CBZ at 96 hpf, this metabolite might, therefore, be interesting to further investigate.

Since HPPH was not detected in the culture medium and embryos/larvae extracts exposed to 250 µM PHE at 120 hpf, a stage when the CYP enzymes should be active [19], we investigated whether PHE is metabolized by zebrafish, using adult zebrafish liver microsomes. To investigate potential species differences, human, rat and rabbit liver microsomes were also included. CBZ was also investigated in this analysis to get a better view on the metabolism of this compound. For both CBZ and PHE, the detection of E-CBZ and HPPH was performed at 240 min in the reaction mix with adult zebrafish liver microsomes. However, the turnover of the parent compounds to these metabolites was much lower than in the other species. For CBZ to E-CBZ, the turnover in zebrafish was 3 times lower than in the rat, 17 times lower than in human and 82 times lower than in rabbit. For the turnover of PHE to HPPH, it was around 2.5 lower than in human, 50 than in the rat and 86 times lower than in the rabbit. This supports the idea that adult zebrafish have a lower ability to metabolize these two compounds into the same metabolites than in humans. However, this study also highlights striking differences between human and species routinely used in developmental toxicology, the rat and rabbit. While rabbit microsomes overestimate the turnover of both compounds compared with humans (5 times higher for CBZ to E-CBZ and 34 times higher for PHE to HPPH), the rat turnover was lower than humans for CBZ to E-CBZ (6 times lower than human) and higher for PHE to HPPH (19 times higher than human). This shows, again, that in-vitro drug metabolism assays are critical when selecting non-clinical species for safety evaluation of pharmaceuticals. This was very recently confirmed by others [67] for mammalian species, but our study shows that adding the zebrafish to the testing battery would also be very useful for pharmaceutical companies, as the turnover rate of PHE to HPPH of the zebrafish was much more similar to human (only 2.5 times lower) than the turnovers of rat and rabbit. Indeed, Chng et al. [68] showed the importance of selecting the relevant toxicological model for cross-species correlation, as they observed species differences in acetaminophen (APAP) metabolism and APAP-induced cytotoxicity. They investigated the bioactivation of APAP to its reactive metabolite, N-acetyl-p-benzoquinone imine (NAPQI). Zebrafish liver microsomes generated the same metabolite, but to a lower extent than human liver microsomes, resulting in no toxicity findings for NAPQI in this species. Distinctive in-vitro metabolite rate formation was also reported between zebrafish and humans for a variety of compounds, such as APAP [68], ibuprofen [69], cisaprid [70], verapamil [70] and diclofenac [71]. Moreover, different metabolite profiles were also observed for clofibric acid [38], testosterone [68,71,72] and ibuprofen [69], where non-human metabolites were found in the reaction mixture with zebrafish liver microsomes. These findings corroborate our earlier statement that human liver microsomes, rather than rat liver microsomes, should be used in the mDarT or the metabolic zebrafish embryo developmental toxicity assay (mZEDTA) when using zebrafish for human risk assessment [30]. Finally, for CBZ and PHE, our investigation at the adult stage also revealed key information for the embryonic and larval stages. E-CBZ was formed after exposure of zebrafish adult liver microsomes to CBZ, albeit at a lower extent than in human, rat and rabbit. In addition, E-CBZ was detected from 96 hpf, indicating the late onset of biotransformation capacity during organogenesis in zebrafish, in accordance with Verbueken et al. [19]. For PHE, while very low concentrations of HPPH were detected after exposure of zebrafish adult liver microsomes to PHE, no clear formation of HPPH was retrieved at the embryonic and larval stage. These findings indicate that either CBZ and PHE are teratogens and do not need bioactivation to exert their developmental toxicity in the zebrafish or other metabolic pathways than in mammals are involved. Further untargeted UHPLC-QTOF-MS investigations could aid in further understanding of the teratogenicity of CBZ and PHE and the potential role of non-CYP mediated pathways.

## 4. Materials and Methods

### 4.1. Adult Zebrafish Housing and Egg Production 

Adult zebrafish (*Danio rerio*), from an in-house wild-type AB line of a year of age, were housed in 60-litre (L) glass aquaria. The fish density was <1 fish/L, and an equal ratio of females to males was ensured. Reverse osmosis water (Barnstead™ Pacific™ RO Water Purification System, Thermo Scientific™, Waltham, MA, USA) in the aquaria was set at 28.5 °C ± 0.3 °C and contained commercial sea salts (Instant Ocean^®^ Sea Salt, Blacksburg, VA, USA) and sodium bicarbonate (Merck, Darmstadt, Germany) to reach a pH of 7.5 ± 0.3 and a conductivity of and 500 ± 40 µS/cm, respectively. Fish were exposed to an automated 14/10 h light/dark cycle. Fish health, as described by Borges et al. [73], and water parameters were checked daily, and the water of the aquaria was renewed when levels of ammonia (NH_3_), nitrite (NO_2_^−^) or nitrate (NO_3_^−^) reached detectable values (NH_3_ < 0.02 mg/L, NO_2_^−^ < 0.3 mg/L and NO_3_^−^ < 12.5 mg/L). During the period of active embryo collection, fish were fed twice a day with Tropical quintet (Montford, The Netherlands) at a rate of 2% of their mean wet weight per feeding. For zebrafish embryos collection, adult zebrafish were transferred in the spawning aquaria on the evening before mating at a density of <1 fish/L. Two bottom nets were present in the spawning aquaria, to prevent adults from eating the embryos. The next morning, the females spawn eggs, after activation of the light cycle and eggs, were allowed to be fertilized for approximately an hour, after which the embryos were collected from the bottom of the tank. Clean embryos were selected, using an Olympus CKX41 microscope with an Olympus U-TV0.5XC-3 lighting and an Olympus 4x/0.16 Uplan APO microscope objective (Olympus Life Science, Shinjuku, Tokyo, Japon) for normal cell division in the first cell cycles (4- to 64-cell stage) [74]. Selected embryos were then randomly transferred to a 48-well plate (Cell culture multiwell plate, 48 wells, clear, Cellstar^®^, Greiner Bio-One, Frickenhausen, Germany) with one embryo per well, filled with embryonic solution (EMS, 0.3 g Instant Ocean^®^ Sea Salt, 0.02 g sodium bicarbonate diluted in 1 L of reverse osmosis water, pH and conductivity adjusted to 7.5 ± 0.3 and 500 ± 40 µS/cm) and kept at 28.5 °C ± 0.3 °C in an incubator (TIN-IN35, Phoenix instrument, Garbsen, Germany) awaiting further steps.

### 4.2. Zebrafish Embryo and Larva Exposure to CBZ, E-CBZ, PHE and HPPH

#### 4.2.1. Test Conditions: Zebrafish Embryos and Larvae Exposure 

Each experiment was performed in two biological replicates (*n* = 2, 20 embryos/replicate) and consisted of a medium control group (EMS), solvent control group(s) (EMS + 0.5% DMSO and/or EMS + 1% DMSO) and four dose groups. A batch of embryos was valid when at least 80% of all eggs were fertilized and mortality/developmental abnormalities of the control groups were lower than 10% throughout the experiment (5¼–120 hpf). Embryos were reared until they reached the desired developmental stage, under the same environmental conditions as the adults (28.5 °C ± 0.3 °C with a 14/10 h light/dark cycle) in the incubator (TIN-IN35, Phoenix instrument, Garbsen, Germany). 

For the parent compounds CBZ (≥99.0% purity, CAS number: 298-46-4, Supelco, Merck, Darmstadt, Germany) and PHE (pharmaceutical primary standard, CAS number: 57-41-0, Supelco, Merck, Darmstadt, Germany), three concentrations were selected (see Supplementary Table S1): a low (31.25 µM), medium (85 µM) and high concentration (250 µM). These concentrations were based on Weigt et al. [35], who showed that the lowest concentration (31.25 µM) affected, combining teratogenic and lethal effects, less than 20% of embryos and around 50% of embryos at the highest (250 µM) concentration, after three days of exposure. For the metabolites, E-CBZ (≥98% purity, CAS number: 36507-30-9, Supelco, Merck, Darmstadt, Germany) and HPPH (98% purity, CAS number: 2784-27-2, Sigma-Aldrich^®^, Merck, Darmstadt, Germany), we only selected one high concentration, 250 µM (see Supplementary Table S1). As the highest concentration, 250 µM, of the parent compounds was known to induce teratogenic effects by 72 hpf in embryos, we therefore expected to also observe teratogenic effects for the metabolites at this concentration. The test compounds were first diluted in DMSO (Sigma Aldrich^®^, Merck, Darmstadt, Germany) and then in EMS with a final percentage of 0.5% DMSO for all concentrations of CBZ, PHE, HPPH and 1% for E-CBZ. A higher percentage of DMSO was used for E-CBZ for the optimum dissolution of E-CBZ in EMS. Immediately after the selection (see Section 4.1), twenty embryos were randomly distributed in each control and test group. Embryos were exposed at the latest at 5 hpf in a final volume of 300 µL medium at a density of a fish per well. 

**Figure 9:**
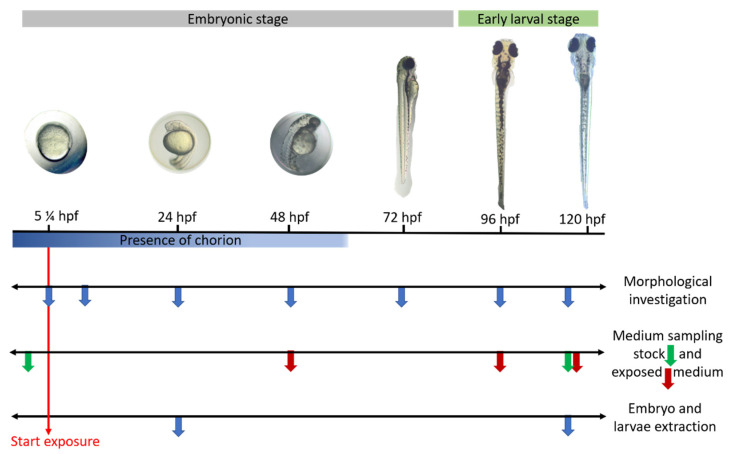
Schematic diagram of the zebrafish embryo exposure experiment. Each vertical-colored arrow represents a time point where the procedure mentioned on the black horizontal axis was carried out. Hpf: hours post fertilization.

The control and test mediums were also kept in the same incubator until the last time point of the experiment. The embryo media were renewed every 48 h, to avoid medium acidification and oxygen deprivation. At each medium change and at the last time point of the experiment (Figure 9), the media exposed to the embryos were pooled and kept at −80 °C for further use (*n* = 3). Moreover, samples from the stock solutions kept at 28.5 °C ± 0.3 °C were also taken at 0 and 120 hpf (*n* = 3), whereas samples were only taken at 96 hpf for the metabolites medium (E-CBZ and HPPH).

In three biological replicates (*n* = 3; 20 embryos/replicate) and at the two desired developmental stages (24 and 120 hpf), embryos/larvae of each test and control group were pooled and then rinsed three times with cold EMS (4 °C) on a mesh (Cell strainer 100 µM Nylon, Steril Falcon^®^, Durham, NC, USA). Embryos collected at 24 hpf were not dechorionated, as the focus of this study was on the potential formation of the mammalian active metabolites. Moreover, it was argued that the chorion was more permeable than previously believed and that dechorionation could increase background malformations during gross morphology investigation [75]. Embryos/larvae were then transferred and kept for five min in cryotubes (Cryo.S™, Greiner Bio-One, Frickenhausen, Germany) filled with 500 µL of cold embryo medium (4 °C), before being snap-frozen in liquid nitrogen and kept, for later use, at −80 °C (Figure 9).

#### 4.2.2. Morphological Evaluation of the Embryos/Larvae over Time

For each biological replicate (*n* = 2), zebrafish embryos were morphologically investigated at 5¼, 10, 24, 48, 72, 96 and 120 hpf (Figure 9) using an Olympus CKX41 microscope with an Olympus U-TV0.5XC-3 lighting and an Olympys 4x/0.16 Uplan APO microscope objective (Shinjuku, Tokyo, Japon) to follow the developmental stages described by Kimmel et al. [74]. The 5¼- and 10-hpf time points served as control steps to identify unfertilized or coagulated eggs that were included unwittingly to the experiment and referenced as coagulated in the 24-hpf morphological sheet (see Supplementary Figure S3). A series of morphological parameters were explored, adapted from Nagel et al. [76], depending on the different time points of interest and described previously by our group [28] (see Supplementary Figure S3). All parameters were scored 0 if normal and 1 if malformed. This binary scale was used to facilitate the scoring and no gradation of the severity of the endpoints was evaluated in this study. As described by Weigt et al. [35], fingerprint endpoints were found when the parameter evaluated followed a concentration-response relationship and was observed in at least 50% of all embryos showing teratogenic effects in all test groups of a test substance.

#### 4.2.3. Extraction of the CBZ and PHE, and the Metabolites, E-CBZ and HPPH, in Embryos and Larvae

The extraction preparation was based on Brox et al. [39] for two developmental stages, 24 and 120 hpf, and performed in triplicate for each test group (*n* = 3, 20 embryos/replicate) (Figure 9). On the day of the extraction procedure, frozen samples (see Section 4.2.1) were thawed on ice and EMS was removed. A volume of 500 µL of acetonitrile (ACN) (purity ≥99.9%, Merck, Darmstadt, Germany) containing the internal standard lamotrigine, LAMO, (≥98% purity, CAS number: 84057-84-1, Sigma-Aldrich, Merck, Darmstadt, Germany) was then added to each tube to reach a final concentration of 0.39 µM, after dilution in HPLC-grade water. Samples were vortexed (VM3, CAT, Germany) for 5 min at a 2000 rotation/min speed, before being placed on ice and ultrasonicated with an Ultrasonic Processor VCX 130 (Sonics, Newtown, CT, USA) fifteen times for 30 s at an 80% amplitude with pause intervals of 30 s. Supernatants of the samples were then centrifuged (Centrifuge 5424 R, Eppendorf, Hamburg, Germany) twice at 4 °C for 15 min at 15,000× *g*, snap-frozen and kept for later use at −80 °C, whereas the pellets were discarded.

### 4.3. In-Vitro Drug Metabolism of CBZ and PHE in Adult Zebrafish, Rat, Rabbit and Human Tissue

#### 4.3.1. Adult Zebrafish Liver Tissue Collection and Microsomal Protein Preparation

Adult zebrafish liver microsomes were prepared from batches of pooled female and male (*n* = 10/batch; 50:50 female:male) adult zebrafish livers of one year of age. After food deprivation of 24 h, the fish were euthanized by being first transferred to cold water (4 °C) for 30 to 60 s, until loss of consciousness, followed by decapitation and rapid destruction of the brain according to the Guidelines for the Euthanasia of Animals of the American Veterinary Medical Association (AVMA). Liver tissues were dissected and rinsed with pre-cooled (4 °C) washing buffer consisting of 1.15% potassium chloride (Supelco, Merck, Darmstadt, Germany) and 10 mM potassium-phosphate buffer (K_3_PO_4_) (Corning^®^ Gentest™, NY, USA) diluted in reverse osmosis water at pH 7.4. Liver tissues were then transferred to a homogenization buffer composed of 1 mM EDTA (Thermo Fisher Scientific, Waltham, MA, USA) and one unit of Halt™ Protease Inhibitor Single-Use Cocktail per 10 mL buffer (Thermo Fisher Scientific, Waltham, MA, USA) and diluted in a washing solution at pH 7.4. The pooled liver tissues of 10 fish were then snap-frozen in liquid nitrogen and kept at −80 °C until further use. The Ethical Committee of Animal Experimentation from the University of Antwerp (Belgium) approved the use of animals in this study (ECD 2018-08). 

The isolation of zebrafish adult liver microsomes was based on Hill et al. [77] and performed as described in Verbueken et al. [18]. Total protein content in the adult zebrafish microsomes samples was determined using the Pierce bicinchoninic acid assay (BCA Assay; Pierce Chemical, Rockford, IL, USA).

#### 4.3.2. Microsomal Incubation with CBZ and PHE

The exposure of adult zebrafish liver microsomes to the anti-epileptic drugs (*n* = 3) was performed in parallel with pooled New Zealand Rabbit Liver microsomes (pooled female donor, L1500, lot number: 1010273) and pooled Sprague–Dawley Rat Liver microsomes (pooled female donor, R1500, lot number: 1110040) purchased from Xenotech, LCC (Tebu-bio, Boechout, Belgium). Moreover, positive and negative controls were included: pooled human liver microsomes (Gibco™, HMMCPL–PL050B, Thermo Fisher Scientific) and Insect Cell Control Supersomes™ (lot number: PL050 C-B, 456201, Corning Incorporated, Corning, NY, USA), respectively. Based on Oziolor et al. [22], microsomal reactions were initiated after adding the substrate solution at a concentration of 100 µM (see Supplementary Table S1) in a mixture of 100 mM K_3_PO_4_ buffer (Corning^®^ Gentest™, NY, USA) of pH 7.4, 5% and 1% of NADPH regenerating system reagents A and B, respectively (Corning, Woburn, MA, USA) and 200 µg/mL of microsomal protein, for a total volume of 500 µL. The reactions were performed at 28.5 ± 0.3 °C until the time point of interest. For the substrate solutions, CBZ and PHE were diluted in 100-mM K_3_PO_4_ buffer with a final concentration of 0.25% DMSO. The reactions were stopped at two time points (5 and 240 min) by sampling 40 µL, in duplicate, of the main microsomal reaction and mixed with 120 µL cold (4 °C) acetonitrile (ACN) (Merck, Darmstadt, Germany) already containing the internal standard (IS), LAMO. The samples were then centrifuged for 10 min at 10,000× *g* to precipitate the proteins, which were denatured in cold ACN. The supernatants were then diluted in 70:30 (*v*/*v*) H_2_O:ACN (high-performance liquid chromatography (HPLC)-grade) with ultrapure H_2_O (Thermo Fisher Scientific (Waltham, MA, USA) to reach a final percentage of 30% ACN and a final concentration of 0.39 µM of LAMO in the samples. An exposure of 240 min was chosen, as metabolization could only be detected from 180 min of incubation with adult zebrafish liver microsomes in our pilot study, for both compounds.

### 4.4. Analytical Investigation

#### 4.4.1. Quantification of the Parent Compounds, CBZ and PHE, in Zebrafish Embryo Culture Medium and Zebrafish Embryos/Larvae Extracts 

For the quantification of CBZ and PHE, zebrafish culture and stock medium (see Section 4.2.1) and zebrafish embryos/larvae extract samples (see Section 4.2.3) from the morphological investigation were analyzed. First, both sample types were processed to reach a final percentage of 30% ACN and a concentration of 0.39 µM of the IS, LAMO. To quantify the concentrations of CBZ and PHE in these samples, calibration curves of 10 concentrations (in duplicate) for CBZ and PHE (12.5–2500 ng/mL) were prepared as well as quality controls in quadruplicate for low (12.5 ng/mL), medium (250 ng/mL) and high (2.5 µg/mL) concentrations (see Supplementary Table S1). Calibration curves and quality controls were prepared in their corresponding reaction mixtures to minimize matrix or extraction process effects. The analytical investigations were performed on an Acquity Ultra Performance Liquid Chromatography (UPLC) with sample manager, binary solvent manager, diode array detector and a triple-quadrupole detector (ACQUITY UPLC-triple quadrupole detector, Waters, Milford, CT, USA) equipped with the Masslynx software (version 4.1). The chromatographic separation of the three analytes (CBZ, PHE, and LAMO) was performed on an Acquity UPLC HSS T3 (2.1 × 100 mm; 1.8 μm) column (Waters, Milford, CT, USA) and elution was conducted with an HPLC-grade mobile phase consisting of water with 0.1% formic acid (A) and ACN containing 0.1% of formic acid (B) (Formic acid 99% ULC/MS grade, CAS 64-18-6, and ACN, CAS 75-05-8, Biosolve, Dieuze, France). Separation was accomplished in 6 min, using a flow rate of 0.5 mL/min. The solvent gradient program was set as follows: 85% A/15% B (0–0.5 min); 85–0% A/15–100% B (0.5–3.3 min); 0% A/100% B (3.3–4.4 min); 0–85% A/100–15% B (4.4–4.5 min); 85% A/15% B (4.5–6 min). The column was set at 40 °C and the injection volume was 10 μL. For the mass spectrometric conditions, the following parameters were used in positive ionization mode: capillarity voltage 3.5 kV, extractor voltage 3 V, cone voltage 35 V, Rf lens 0.1 V. The source temperature was set at 120 °C and the desolation temperature at 450 °C. The desolvation gas flow was fixed at 1000 L/h and cone gas flow at 50 L/h. The analytes were quantified via multiple reaction monitoring for CBZ, PHE and the IS; LAMO and the chosen quantifier and qualifier transitions for each analyte are presented in Table 5. The limit of quantification, linearity, matrix effects and specificity of the method developed for each compound were validated based on the European Medicines Agency (EMA) guidelines for bioanalytical method validation and the study from Kislyuk et al. [37]. In brief, the lower limits of quantification (LLOQ) for CBZ and PHE were 3.5 ng/mL and 1.75 ng/mL respectively (see Supplementary Table S1). No matrix effect was observed in either the culture medium samples nor the zebrafish embryos/larvae extract samples.

#### 4.4.2. Quantification of the Metabolites, E-CBZ and HPPH in Zebrafish Embryo Culture Medium, Zebrafish Embryos/Larvae Extracts and Microsomal Reaction Samples

For the quantification of the metabolites E-CBZ and HPPH, the samples processed were the microsomal incubation reactions (see Section 4.3.2), the zebrafish culture and stock medium (4.2.1) and the zebrafish embryos/larvae extracts (see Section 4.2.3). Samples from the different experiments were also prepared, as for the parent compounds, to reach a final percentage of 30% ACN and a concentration of 0.39 µM of the IS, LAMO. The samples were then stored at −80 °C until use. To quantify the concentrations of E-CBZ and HPPH in these samples, standard curves (in duplicate) of 10 concentrations for E-CBZ (0.5–400 ng/mL; see Supplementary Table S1) and HPPH (2–1600 ng/mL; see Supplementary Table S1) were prepared, as were quality controls. The concentrations selected as quality controls for E-CBZ and HPPH were as follow (see Supplementary Table S1): low (1.5 ng/mL E-CBZ/6 ng/mL HPPH), medium (20 ng/mL E-CBZ/80 ng/mL HPPH), high (75 ng/mL; E-CBZ/300 ng/mL HPPH) and very high (250 ng/mL E-CBZ/1000 ng/mL HPPH) concentrations. Calibration curves and quality controls were prepared in their corresponding reaction mixtures to minimize matrix or extraction-process effects. For these two metabolites, an instrumental method was developed and optimized using a Shimadzu UPLC-MS-8050 triple-quadrupole system. Chromatography separation was carried out on a Phenomenex Kinetex™ Biphenyl (50 × 2, 1 mm, 1.7 μm) column set at 40 °C and an HPLC-grade mobile phase consisting of water with 0.1% formic acid (A) and methanol containing 0.1% of formic acid (B.) (Formic acid 99% ULC/MS grade, CAS 64-18-6, ACN CAS 75-05-8 and Methanol, CAS 67-56-1, Biosolve, Dieuze, France). The chromatographic separation of the three analytes (E-CBZ, HPPH and LAMO) was also accomplished in 6 min, using a flow rate of 0.5 mL/min with an injection volume of 10 μL. The mobile phase was carried out in a gradient mode 75–50% A/25–50% B, with the following steps: 75% A/25% B (0–1 min); 75–50% A/25–50% B (1–3 min); 50% A/50% B (3–4 min), then returned to the initial value with time for equilibration. Analysis was performed with an ionizing voltage of 4000 V. The interface temperature was set at 300 °C and the desolvation line temperature was fixed at 250 °C, with ultrahigh-purity nitrogen for the drying gas (10 L/min), nebulizer gas (3 L/min) and heating gas (10 L/min). The quantification of the analytes was carried out via multiple reaction monitoring (MRM) in positive ion mode. The quantifier and qualifier transitions chosen for E-CBZ, HPPH and LAMO are presented in Table 5. As for the parent compounds, the limits of quantification, linearity, matrix effects and specificity of the method developed for each metabolite were validated based on the EMA guidelines for bioanalytical method validation and the study from Kislyuk et al. [37]. The LLOQ were for E-CBZ 1.5 ng/mL and 6 ng/mL for HPPH (see Supplementary Table S1). No matrix effects were observed for all samples investigated.

### 4.5. Calculated and Statistical Data

All statistical analyses were conducted using GraphPad Prism 8 (San Diego, CA, USA) and *p*-values < 0.05 were considered to indicate a statistically significant difference between the control and test groups.

As a binary scale was used for the morphological investigation of zebrafish embryos and larvae (no malformation: 0; malformation: 1), we analyzed the data using non-parametric Fisher’s exact tests. The potential effect from the controls was first compared with the embryos/larvae only exposed to EMS. When no statistical differences were observed, the data from the exposed groups were compared with their corresponding control groups at each time point. To compare the continuous data, such as the quantified compound concentrations in the collected culture medium, embryo extract samples and microsomal incubation reactions, normal distribution and homogeneity of variances were first investigated by Kolmogorov–Smirnov’s tests and Brown–Forsythe’s tests, respectively. If the data did not meet the latter’s characteristics, data were log-transformed. One-way ANOVA tests were then conducted to compare the groups, followed by Tukey’s multiple comparisons test for the quantified concentration in culture/stock mediums and zebrafish embryos/larvae extract and Dunnett’s multiple comparison post-hoc tests to detect differences for the microsomal investigation. All the different groups in the microsomal incubation investigation were compared with the liver microsomes of our species of interest, the adult zebrafish.

## 5. Conclusions and Future Perspectives

This study showed, first, that extending the exposure window for CBZ and PHE to 120 hpf in the ZEDTA affects organs that are developed later in organogenesis. As such, a more complete view on the teratogenic effects of these compounds was obtained, which can be useful in view of the AOP framework. Second, we noted that the direct exposure of zebrafish embryos to E-CBZ and HPPH, which cause teratogenicity in mammals, did not cause morphological abnormalities, whereas the parent compounds, CBZ and PHE, induced teratogenicity, even from 48 hpf onwards. As zebrafish larvae only generated E-CBZ from 96 hpf onwards and no HPPH was formed when exposing zebrafish embryos to PHE, it is safe to conclude that these metabolites do not play a role in PHE- and CBZ-induced teratogenicity in zebrafish. From a broader perspective, our study and earlier data on the ontogeny of CYPs in zebrafish suggest that the CYP-mediated bioactivation of xenobiotics is not the key driver for teratogenicity in zebrafish. Finally, our in-vitro drug metabolism assays with human, rat, rabbit, and zebrafish adult liver microsomes showed prominent species differences in metabolic rate for CBZ and PHE. Overall, the zebrafish seems a poor metabolizer of CBZ, whereas rabbit was an ultrarapid metabolizer. For PHE, the zebrafish metabolic rate was closer to human than that of rat or rabbit. This shows again that no preclinical species is the ideal translational model for human drug metabolism and that this is compound-specific. Therefore, we recommend including the zebrafish in the in-vitro drug metabolism testing battery when this species is considered for evaluating the safety of pharmaceuticals under development.

From our study, we cannot exclude a potential role of other metabolic pathways than CYPs in the teratogenicity of CBZ and PHE. This should be further investigated with untargeted LC-MS. Although time and effort consuming, UHPLC-QTOF-MS can aid in verifying whether CBZ and PHE are teratogenic by themselves or whether other metabolites, albeit different than mammalian ones, are involved. As such, the potential role of other Phase I enzymes than CYPs or Phase II enzymes in zebrafish developmental toxicity can be elucidated. Such insights will help to fully understand the metabolic capacity of the zebrafish embryo model and in the development of accurate AOPs.

## Figures and Tables

**Table 1:** Morphological evaluation of zebrafish larvae at 120 hpf in the medium control group, solvent control groups (EMS + 0.5% DMSO and EMS + 1% DMSO) and test groups (CBZ—E-CBZ). Only parameters showing abnormalities are mentioned in the tables. EMS: embryo solution; DMSO: dimethyl sulfoxide; Hpf: hours post fertilization; Exp: experiment. * *p* < 0.05, ** *p* ≤ 0.01, **** *p* ≤ 0.0001.

				Deviation Tail		Oedema			Malformation Head				
120 hpf Experiment CBZ/E-CBZ	#	Coagulation	No Hatching	Curve	Tissue Deviation	Head	Pericardium	Yolk	Deviation Shape Head	Deviation Mouth	Deviation Eye	Deviation Pigmentation	Swim Bladder Not Inflated
EMS	1			2/20	2/20				2/20				1/20
	2												
EMS 0.5% DMSO	1		1/20		1/20				1/20				
	2						1/20				1/20		2/20
EMS 1% DMSO	1				1/20				2/20				
	2				1/20								1/20
CBZ 31.25 µM	1												8/20 **
	2												2/20
CBZ 85 µM	1				1/20								7/20 **
	2	2/20		1/18	1/18				3/18	2/18	1/18		2/18
CBZ 250 µM	1		5/20 *		2/20		5/20 *	3/20	4/20		5/20 *	19/20 ****	16/20 ****
	2		1/20	2/20	4/20		3/20		3/20	3/20	8/20 **	18/20 ****	11/20 **
E-CBZ 250 µM	1					1/20	1/20	1/20			1/20		3/20
	2			1/20						1/20	1/20		6/20 *

Ratios depicted in bold are statistically significant compared to the solvent control group.

**Table 2:** Morphological evaluation of zebrafish larvae at 120 hpf in the medium control group, solvent control group (EMS + 0.5% DMSO) and test groups (PHE—HPPH). Only parameters showing abnormalities are mentioned in the tables. EMS: embryo solution; DMSO: dimethyl sulfoxide; Hpf: hours post fertilization; Exp: experiment. * *p* < 0.05, *** *p* ≤ 0.001, **** *p* ≤ 0.0001.

			Deviation Tail			Oedema			Malformation Head			
120 hpf Experiment PHE/HPPH	#	No Hatching	Elbow	Curve	Tissue Deviation	Head	Pericardium	Yolk	Deviation Shape Head	Deviation Eye	Deviation Pigmentation	Swim Bladder Not Inflated
EMS	1										1/20	1/20
	2											
EMS 0.5% DMSO	1				2/20				2/20	1/20	1/20	1/20
	2			1/20	1/20				1/20	1/20		2/20
PHE 31.25 µM	1									2/20	2/20	4/20
	2			1/20							4/20	2/20
PHE 85 µM	1			3/20	2/20	1/20			4/20	2/20	2/20	6/20
	2		1/20	4/20	2/20	2/20			3/20	3/20	2/20	13/20 ***
PHE 250 µM	1	1/20		7/20 *	1/20	1/20	1/20	1/20	2/20	3/20	3/20	16/20 ****
	2	1/20	2/20	5/20	1/20	1/20	1/20		3/20	4/20	7/20 *	15/20 ****
HPPH 250 µM	1			2/20	1/20					2/20		5/20
	2				1/20							6/20

Ratios depicted in bold are statistically significant compared to the solvent control group.

**Table 3:** Percentage of quantified compound concentration in culture medium (CBZ–E-CBZ) exposed to zebrafish embryos/larvae relatively to the nominal concentration at the different time points. Each percentage represents the average of the three replicates. Hpf: hours post fertilization; [X]: concentration.

Nominal [X]	31.25 µM				85 µM				250 µM				
Time Points	0 hpf	48 hpf	96 hpf	120 hpf	0 hpf	48 hpf	96 hpf	120 hpf	0 hpf	48 hpf	96 hpf		120 hpf
Compound	CBZ				CBZ				CBZ			E-CBZ	CBZ
Stock	95.6 ± 2.0%			101.6 ± 7.7%	81.7 ± 18.7%			90.4 ± 6.6%	92.1 ± 5.3%				98.4 ± 4.6%
Culture medium		95.4 ± 16.2%	101.2 ± 21.7%	109.6 ± 5.4%		110.0 ± 4.8%	116.8 ± 4.7%	113.9 ± 4.5%		113.4 ± 14.1%	114.2 ± 2.6%	117.3 ± 9.3%	111.5 ± 2.6%

**Table 4:** Percentage of quantified compound concentration in culture medium (PHE–HPPH) exposed to zebrafish embryos/larvae relatively to the nominal concentration at the different time points. Each percentage represents the average of the three replicates. Hpf: hours post fertilization; [X]: concentration.

Nominal [X]	31.25 µM				85 µM				250 µM				
Time Points	0 hpf	48 hpf	96 hpf	120 hpf	0 hpf	48 hpf	96 hpf	120 hpf	0 hpf	48 hpf	96 hpf		120 hpf
Compound	PHE				PHE				PHE			HPPH	PHE
Stock	53.3 ± 23.3%			62.01 ± 5.25%	86.4 ± 47.2%			64.5 ± 1.2%	24.7 ± 3.4%				30.6 ± 7.9%
Culture medium		80 ± 4.8%	84.5 ± 8.4%	81.2 ± 11.1%		79.1 ± 19.6%	64.8 ± 8.7%	64.2 ± 10.7%		33.2 ± 6.6%	43.3 ± 8.4%	89.9 ± 15.5%	33.4 ± 1.2%

**Table 5:** Quantifier and qualifier transitions followed on the UPLC triple-quadrupole systems for each compound. CBZ: carbamazepine; PHE: phenytoin; LAMO: lamotrigine; E-CBZ: carbamazepine-10,11-epoxide; HPPH: 5-(4-hydroxyphenyl)-5-phenylhydantoin.

LC-MS System	Compound	Quantifier Transition	Qualifier Transition
ACQUITY UPLC-triple quadrupole system	CBZ	237 → 194	237 → 165
	PHE	253 → 182	253 → 104
	LAMO	256 → 211	256 → 108
Shimadzu UPLC-MS-8050 triple quadrupole system	E-CBZ	253.1 → 210.2	253.1 → 180.3
	HPPH	268.9 → 120.1	268.9 → 104.2
	LAMO	255.9 → 109.1	255.9 → 211.1

## Data Availability

Not applicable.

## Supplementary Materials

The following are available online at https://www.mdpi.com/article/10.3390/ijms222312696/s1.

## Abbreviations

ACN	acetonitrile
AOP	adverse outcome pathway
CBZ	carbamazepine
CYP	cytochrome P450
DMSO	dimethyl sulfoxide
E-CBZ	carbamazepine-10,11-epoxide
ECVAM	European Centre for the Validation of Alternative Methods
EDTA	ethylenediaminetetraacetic acid
EMA	European Medicines Agency
EMS	embryo solution
H	hours
Hpf	hours post fertilization
H_2_O	water
HPLC	high-performance liquid chromatography
HPPH	5-(4-hydroxyphenyl)-5-phenylhydantoin
IS	internal standard
L	liters
LAMO	lamotrigine
LC-MS	liquid chromatography-mass spectrometry
LLOQ	lower limit of quantification
mDarT	metabolic *Danio rerio* test
Min	minutes
mL	millilitres
mZEDTA	metabolic zebrafish embryo developmental toxicity assay
PHE	phenytoin
µL	microliters
Xg	time gravity
%	percentage
