# Supplementary material for: Developmental Toxicity and Biotransformation of Two Anti-Epileptics in Zebrafish Embryos and Early Larvae
Source: Int J Mol Sci. 2021 Nov 24;22(23):12696. doi: 10.3390/ijms222312696 (PMC8657848; doi:10.3390/ijms222312696)
Supplement: Supplementary file 1 [file ijms-22-12696-s001.zip › ijms-1440720-supplementary.pdf]

International Journal of
Molecular Sciences

MDPI

Supplementary data

(**A**)

24 hpf - Experiment CBZ/E-CBZ	#	Body parts indistinguishable	Deviation tail	Oedema		Cardiovasular system	Malformation head
			Tissue deviation	Pericard	Yolk	Malformation heart	Deviating shape of head
EMS	1						
	2						
EMS 0.5% DMS0	1						
	2	1/20					
EMS 1% DMS0	1						
	2					1/20	1/20
CBZ 31.25 µM	1						
	2				1/20		
CBZ 85 µM	1						
	2		1/20	1/20			
CBZ 250 µM	1						
	2				1/20		
E-CBZ 250 µM	1						
	2						

(**B**)

48 hpf - Experiment CBZ/E-CBZ	#	Body parts unrecognizable	Hatching	Deviation tail		Malformation head	
				Curve	Tissue deviation	Deviating shape of head	Deviation eye
EMS	1		2/20				
	2		4/20				
EMS 0.5% DMS0	1		3/20				
	2	1/20	10/20			1/20	
EMS 1% DMS0	1		3/20				
	2		14/20				
CBZ 31.25 µM	1		7/20				
	2		12/20				
CBZ 85 µM	1		7/20		1/20		
	2		**2/20 *** **		2/20		
CBZ 250 µM	1		1/20				
	2		9/20	2/20	1/20		1/20
E-CBZ 250 µM	1		8/20		1/20		
	2		15/20				

(**C**)

72 hpf - Experiment CBZ/E-CBZ	#	Coagulation	Body parts indistinguishable	No hatching	Deviation tail		Oedema	Malformation head		Swim bladder not inflated
					Curve	Tissue deviation	Pericard	Deviating shape of head	Deviation eye	
EMS	1				1/20	1/20		1/20		13/20
	2									10/20
EMS 0.5% DMS0	1									11/20
	2		1/20		1/20			1/20	2/20	9/20
EMS 1% DMS0	1									9/20
	2					1/20		1/20		8/20
CBZ 31.25 µM	1				1/20					10/20
	2									9/20
CBZ 85 µM	1				1/20					10/20
	2	1/20				1/19			2/19	6/19
CBZ 250 µM	1			**7/20 ** **		1/20				**18/20 * **
	2			**8/20 ** **		3/20	3/20		1/20	12/20
E-CBZ 250 µM	1									9/20
	2							1/20	1/20	6/20

(**D**)

96 hpf - Experiment CBZ/E-CBZ	#	Coagulation	No hatching	Deviation tail		Oedema	Oedema	Malformation head		Deviating pigmentation	Swim bladder not inflated
				Curve	Tissue deviation	Pericard	Yolk	Deviating shape of head	Deviation eye		
EMS	1			1/20	1/20			2/20			6/20
	2										4/20
EMS 0.5% DMS0	1										7/20
	2		1/20			1/20	1/20	1/20	1/20		3/20
EMS 1% DMS0	1										8/20
	2										5/20
CBZ 31.25 µM	1										6/20
	2				1/20						4/20
CBZ 85 µM	1			1/20	2/20			2/20			9/20
	2	1/20		1/19	3/19			2/19			5/19
CBZ 250 µM	1		**5/20 ***	1/20	**5/20 ***			**5/20 ***		**13/20 *******	**16/20 *****
	2		4/20	1/20	4/20	2/20	1/20	2/20		**15/20 *******	8/20
E-CBZ 250 µM	1										10/20
	2			1/20	1/20			2/20	1/20		4/20

Figure S1.(**A**): CBZ/E-CBZ morphological replicates – Time point 24 hpf, (**B**): CBZ/E-CBZ morphological replicates – Time point 48 hpf; (**C**): CBZ/E-CBZ morphological replicates – Time point 72 hpf; (**D**): CBZ/E-CBZ morphological replicates – Time point 96 hpf

(**A**)

48 hpf - Experiment PHE/HPPH	#	Hatching
EMS	1	10/20
	2	9/20
EMS 0.5% DMS0	1	13/20
	2	13/20
PHE 31.25 µM	1	9/20
	2	11/20
PHE 85 µM	1	6/20
	2	10/20
PHE 250 µM	1	**0/20 *******
	2	**4/20 *****
HPPH 250 µM	1	15/20
	2	15/20

(**B**)

72 hpf - Experiment PHE/HPPH	#	No hatching	Deviation tail		Malformation head	Deviating pigmentation	Swim bladder not inflated
			Curve	Tissue deviation	Deviation eye		
EMS	1						10/20
	2						7/20
EMS 0.5% DMS0	1				1/20		10/20
	2		1/20				7/20
PHE 31.25 µM	1					3/20	13/20
	2						10/20
PHE 85 µM	1			1/20		2/20	13/20
	2		3/20	3/20		4/20	13/20
PHE 250 µM	1	4/20	1/20	1/20	3/20	2/20	14/20
	2	1/20			4/20	**5/20 ***	**17/20 *****
HPPH 250 µM	1				1/20		11/20
	2		1/20		1/20		10/20

(**C**)

96 hpf - Experiment PHE/HPPH	#	No hatching	Deviation tail		Oedema		Malformation head		Deviating pigmentation	Swim bladder not inflated
			Curve	Tissue deviation	Head	Pericard	Deviating shape of head	Deviation eye		
EMS	1								1/20	4/20
	2									4/20
EMS 0.5% DMS0	1			1/20			1/20		1/20	5/20
	2		2/20				1/20			1/20
PHE 31.25 µM	1							2/20	3/20	9/20
	2								4/20	**9/20 ****
PHE 85 µM	1			4/20			3/20	2/20	1/20	11/20
	2		5/20	1/20			2/20	3/20	2/20	**12/20 *****
PHE 250 µM	1	1/20	1/20	1/20		1/20	2/20		3/20	**18/20 ******
	2	1/20	3/20		1/20		1/20	4/20	**5/20 ***	**14/20 ******
HPPH 250 µM	1			1/20			1/20			5/20
	2			1/20						6/20

Figure S2.(**A**): PHE/HHPH morphological replicates – Time point 48 hpf; (**B**): PHE/HHPH morphological replicates – Time point 72 hpf; (**C**): PHE/HHPH morphological replicates – Time point 96 hpf

Table S1. List of molar and mass concentrations used for the different experiments per compounds. CBZ: carbamazepine; E-CBZ: carbamazepine-10,11-epoxide; PHE: phenytoin; HPPH: 5-(4-hydroxyphenyl)-5-phenylhydantoin; LAMO: lamotrigine

Compound	Molar concentration (µM)	Mass concentration	Samples
CBZ	0.02	3.5 ng/ml	LLOQ
	0.05	12.5 ng/ml	Standard curve - Quality control (Low)
	1.06	250 ng/ml	Quality control (Medium)
	10.5	2.5 µg/ml	Standard curve - Quality control (High)
	31.25	7.38 µg/ml	Samples – embryo exposure
	85	20 µg/ml	Samples – embryo exposure
	100	23.62 µg/ml	Samples – microsomes exposure
	250	59 µg/ml	Samples – embryo exposure
E-CBZ	0.002	0.5 ng/ml	Standard curve
	0.006	1.5 ng/ml	Quality control (Low) - LLOQ
	0.08	20 ng/ml	Quality control (Medium)
	0.3	75 ng/ml	Quality control (High)
	1	250 ng/ml	Quality control (Very high)
	1.59	400 ng/ml	Standard curve
	250	63 µg/ml	Samples – embryo exposure
PHE	0.01	1.75 ng/ml	LLOQ
	0.05	12.5 ng/ml	Standard curve - Quality control (Low)
	1.0	250 ng/ml	Quality control (Medium)
	10	2.5 µg/ml	Standard curve - Quality control (High)
	31.25	7.88 µg/ml	Samples – embryo exposure
	85	21.4 µg/ml	Samples – embryo exposure
	100	25.23 µg/ml	Samples – microsomes exposure
	250	63 µg/ml	Samples – embryo exposure
HPPH	0.007	2 ng/ml	Standard curve
	0.02	6 ng/ml	Quality control (Low) - LLOQ
	0.3	80 ng/ml	Quality control (Medium)
	1.12	300 ng/ml	Quality control (High)
	3.73	1000 ng/ml	Quality control (Very high)
	5.97	1600 ng/ml	Standard curve
	2 50	67 µg/ml	Samples – embryo exposure
LAMO	0.39	100 ng/ml	Samples – internal standard

Figure S3. Parameters evaluated during morphological investigation at the 24, 48, 72, 96, 120 hours post fertilization time points

Time point	Parameters																										
24 hpf	Pre-processing			separate deviations of morphology																							
				deviation tail			Oedema				blood accumulation					Mal-formation yolk	Splitt yolk + extension	Cardiovasular system				Malformation head				deviating pigmentation	Non-detach-ment tail
	Coa-gulation	body parts indistinguishable	body parts unrecognizable	elbow	Curve	tissue devi-ation	head	Pericard	Yolk	Yolk extension /tail	tail	head	heart	Yolk	Yolk exten-sion/tail			*Mal-formation heart*	*no blood circulation in tail*	*disturbed blood circulation in tail*	*heartbeat absent*	deviating shape of head	deviation ear	deviation mouth	deviation eye		

Time point	Parameters																											
48 hpf	Pre-processing				separate deviations of morphology																							
					deviation tail			Oedema				blood accumulation					Mal-formation yolk	Splitt yolk + extension	Cardiovasular system				Malformation head				deviating pigmentation	Non-detach-ment tail
	Coa-gulation	body parts indistinguishable	body parts unrecognizable	Hatching	elbow	Curve	tissue devi-ation	head	Pericard	Yolk	Yolk extension /tail	tail	head	heart	Yolk	Yolk exten-sion/tail			*Mal-formation heart*	*no blood circulation in tail*	*disturbed blood circulation in tail*	*heartbeat absent*	deviating shape of head	deviation ear	deviation mouth	deviation eye		

Time point	Parameters																															
72 hpf	Pre-processing				separate deviations of morphology																											
					deviation tail			Oedema				blood accumulation					Malformation pectoral fin				Mal-for-mation yolk	Cardiovasular system				Malformation head				deviating pigmentation	Non-detach-ment tail	swim bladder not inflated
	Coa-gulation	body parts indistinguishable	body parts unrecognizable	no hatching	elbow	Curve	tissue deviation	head	Pericard	Yolk	Yolk extension/tail	tail	head	heart	Yolk	Yolk exten-sion/tail	*missing fin (left)*	*missing fin (right)*	*curved fin (left)*	*curved fin (right)*		*Mal-formation heart*	*no blood circulation in tail*	*disturbed blood circulation in tail*	*heartbeat absent*	deviating shape of head	deviation ear	deviation mouth	deviation eye			

Time point	Parameters																															
96 hpf	Pre-processing				separate deviations of morphology																											
					deviation tail			Oedema				blood accumulation					Malformation pectoral fin				Mal-for-mation yolk	Cardiovasular system				Malformation head				deviating pigmentation	Non-detach-ment tail	swim bladder not inflated
	Coa-gulation	body parts indistinguishable	body parts unrecognizable	no hatching	elbow	Curve	tissue deviation	head	Pericard	Yolk	Yolk extension/tail	tail	head	heart	Yolk	Yolk exten-sion/tail	*missing fin (left)*	*missing fin (right)*	*curved fin (left)*	*curved fin (right)*		*Mal-formation heart*	*no blood circulation in tail*	*disturbed blood circulation in tail*	*heartbeat absent*	deviating shape of head	deviation ear	deviation mouth	deviation eye			

Time point	Parameters																															
120 hpf	Pre-processing				separate deviations of morphology																											
					deviation tail			Oedema				blood accumulation					Malformation pectoral fin				Mal-for-mation yolk	Cardiovasular system				Malformation head				deviating pigmentation	Non-detach-ment tail	swim bladder not inflated
	Coa-gulation	body parts indistinguishable	body parts unrecognizable	no hatching	elbow	Curve	tissue deviation	head	Pericard	Yolk	Yolk extension/tail	tail	head	heart	Yolk	Yolk exten-sion/tail	*missing fin (left)*	*missing fin (right)*	*curved fin (left)*	*curved fin (right)*		*Mal-formation heart*	*no blood circulation in tail*	*disturbed blood circulation in tail*	*heartbeat absent*	deviating shape of head	deviation ear	deviation mouth	deviation eye			
